# Contact structures in the poultry industry in Great Britain: Exploring transmission routes for a potential avian influenza virus epidemic

**DOI:** 10.1186/1746-6148-4-27

**Published:** 2008-07-23

**Authors:** Jennifer E Dent, Rowland R Kao, Istvan Z Kiss, Kieran Hyder, Mark Arnold

**Affiliations:** 1 Centre for Epidemiology and Risk Analysis, VLA, New Haw, Addlestone, Surrey, KT15 3NB, UK; 2 Institute of Comparative Medicine, Faculty of Veterinary Medicine, University of Glasgow, Glasgow, G61 1QH, UK; 3 Department of Mathematics, University of Sussex, Brighton, BN1 9RF, UK

## Abstract

**Background:**

The commercial poultry industry in United Kingdom (UK) is worth an estimated £3.4 billion at retail value, producing over 174 million birds for consumption per year. An epidemic of any poultry disease with high mortality or which is zoonotic, such as avian influenza virus (AIV), would result in the culling of significant numbers of birds, as seen in the Netherlands in 2003 and Italy in 2000. Such an epidemic would cost the UK government millions of pounds in compensation costs, with further economic losses through reduction of international and UK consumption of British poultry. In order to better inform policy advisers and makers on the potential for a large epidemic in GB, we investigate the role that interactions amongst premises within the British commercial poultry industry could play in promoting an AIV epidemic, given an introduction of the virus in a specific part of poultry industry in Great Britain (GB).

**Results:**

Poultry premises using multiple slaughterhouses lead to a large number of premises being potentially connected, with the resultant potential for large and sometimes widespread epidemics. Catching companies can also potentially link a large proportion of the poultry population. Critical to this is the maximum distance traveled by catching companies between premises and whether or not between-species transmission could occur within individual premises. Premises closely linked by proximity may result in connections being formed between different species and or sectors within the industry.

**Conclusion:**

Even quite well-contained epidemics have the potential for geographically widespread dissemination, potentially resulting in severe logistical problems for epidemic control, and with economic impact on a large part of the country. Premises sending birds to multiple slaughterhouses or housing multiple species may act as a bridge between otherwise separate sectors of the industry, resulting in the potential for large epidemics. Investment into further data collection and analyses on the importance of industry structure as a determinant for spread of AIV would enable us to use the results from this study to contribute to policy on disease control.

## Background

The GB commercial poultry industry is an important industry to the British government, the consumer and farmers alike. Worth an estimated £3.4 billion at retail value, producing over 174 million birds for consumption per year [1], poultry diseases are of widespread interest, both from the point-of-view of understanding different poultry farming methods [2-4], and in terms of studying the potential impact of different diseases on poultry [5-7]. However, our knowledge of how poultry farms in GB are connected to each other by the movement of people and equipment is more limited. This is essential for effective prevention and control for potential outbreaks of diseases transmitted by the movement of people and equipment between farms within the commercial poultry industry. Diseases spread in such a way include avian influenza viruses (AIV), Newcastle disease virus (NDV), *Salmonella *and *Campylobacter *species. Motivated by recent outbreaks of AIV in the UK and across the world [8], we consider here how AIV may transmit between poultry farms in GB by the movement of people and equipment.

Avian influenza viruses (AIV) were first described in the late nineteenth century by Perroncito [9]. They are highly contagious viral infections that can affect avian species as well as other species such as pigs [7], cats [10] and humans [11]. High pathogenic strains of the virus (HPAI) have potentially high transmissibility and high mortality rates in poultry [12-14]. Although low pathogenic strains of the virus (LPAI) have a lower mortality rate, this renders them harder to detect, increasing the chances of silent spread. Furthermore, the H5 and H7 subtypes of LPAI have the ability to mutate into high-pathogenic strains as seen in 1999 in Italy, when H7N1 mutated from LPAI to HPAI [15]. In recent years, the H5N1 strain of the virus has gradually spread westward in countries located between Hong Kong (2003) and GB (2007) and it is likely that AIV is endemic in poultry in many parts of the world [16].

In previous studies, AIV outbreak data from other countries have been analysed and models have been developed to describe the spread of the virus [13,16,17]. Recent publications have concentrated on the development of simulation models [8,17] but have not described in detail the contact structures within the industry over which transmission could occur. As it is unclear to what extent transmission parameters from other countries can be applied to models that include detailed industry structure, we use an analytical approach that considers the importance of the contact structures that occur within the poultry industry, with respect to the potential for disease transmission. In the absence of robust estimates of transmission parameters, this approach can be used to identify combinations of parameters that could result in a large epidemic, and critically, under those scenarios identifying key demographic features which lead into determining when infectious diseases may spread, as has previously been done in analyses of the sheep and cattle industries [18-20].

In this study, the structure of the poultry industry in GB is investigated using the poultry network database collected and maintained by the Department for Environment, Food and Rural Affairs (Defra). While there have been no recent large outbreaks of AIV in GB, HPAI, LPAI, NDV, *Campylobacter *and *Salmonella *are all transmitted via the faeco-oral route, and so we infer likely routes of transmission for AIV by the transmission of these diseases in prior outbreaks. The presence of slaughterhouse personnel or equipment on a premises during depopulation has been implicated as a risk factor for infection of *Campylobacter *to remaining birds [21]. Slaughterhouses have also been implicated as a key checkpoint for the detection of pathogens such as *Campylobacter *[22] and *Salmonella *[23,24], and in case of poor biosecurity could also result in the spread of pathogens between premises where dirty equipment is reused. Catching companies (teams of people that catch birds for slaughter) have also been implicated in *Campylobacter *transmission [25], and within company spread includes fomite transmission as well as transmission via personnel, which is considered a major route of transmission of avian infection [2,26]. In addition, "local spread" may be important. Local spread has been identified in transmission of AIV between poultry flocks in the Netherlands [13,27] and environmental factors were highlighted by the literature review as being a potentially important factor for transmission of avian influenza virus between farms [2,6,12]. Such factors include the presence and circulation of wild birds. In this study we considered the effects of the presence and circulation of wild birds on the transmission of avian influenza in the poultry network in GB only in the context of "local transmission", i.e. multiple premises infected from the same wildlife source. By considering associations amongst sub-populations defined by their interactions, e.g. associations with the same catching companies, slaughterhouses, common ownership or by "local spread", we determine the extent to which industry structures might influence the demographic and geographic extent of a potential AIV epidemic in the GB poultry industry. Improvement of our understanding of how poultry premises are potentially connected will also identify where further data collection is necessary.

## Results

### Network construction and properties

The analysis of the poultry industry was restricted to the principle commercial species, i.e. to premises housing turkeys, chickens, ducks and geese. Premises housing fewer than 50 birds are not included in the analysis as such premises are not required to register their birds. Premises with missing location data are also excluded from the analyses. Contact mechanisms between premises were categorised into people, fomites, vehicles and environmental factors, as shown in Table 1[2,15,21,25,28]. These were then organised into their relevance to sub-populations within the poultry industry that may connect poultry premises that they are associated with. These sub-populations were identified as:

**Table 1:** Potential between farm transmission routes of avian influenza virus.

**Vehicles**	**People**	**Fomites**	**Environment**
Litter disposal	Catchers and Thinners	Catching equipment	Wildfowl

Catching	Drivers	Containers	Water and Feed

Disposal and replacement	Cleaning teams	Pallets	Airborne (dust)

Cleaning	Artificial Insemination Teams	Culling equipment	Flying insects

Dead bird collection	Area Managers	Workman's clothes	Game birds (shows)

Imports	Farm staff	Dead bird collecting	

Hatching egg collection	Dead bird collectors	Holding station	

Feed delivery	Vets	Raw feed Material	

Visiting			

Vaccination			

Farm			

Transmission routes are broken down into sub-categories, which are then ranked in order of potential risk of acting as a transmission route of disease, from most important to least important.

▪ Slaughterhouses, whose vehicles are used to collect birds from farms before slaughter

▪ Catching companies responsible for catching birds before they go to slaughter

▪ Egg collectors who visit multiple farms in one shift

▪ Feed companies responsible for delivering feed to premises

▪ Multi-site companies, whose personnel and vehicles may visit multiple sites belonging to the same company

▪ Farms that are geographically close

Feed lorries and egg collectors have not been included here as data on these potential transmission routes was not available for analysis at the time of study. Contact data for the remaining transmission mechanisms were used to construct representations of contact structures occurring within the commercial poultry industry in GB. The collection of contact data was targeted at commercial poultry premises housing a minimum of 1000 birds, as smaller premises are more likely to catch and slaughter their own birds. From a target population of approximately 9075 premises (the number of premises housing >999 birds according to the GB poultry register), a sample of 4441 poultry premises was included and potentially infectious links between premises could occur as a result of premises using the same slaughterhouse, catching company, or belonging to the same multi-site company. Local spread was also included to represent environmental transmission. The sample used was taken from the poultry network database, which has been designed to show the links and movements between premises that either send birds to slaughter, use catching companies or belong to a multi-site company. The database was not designed to give population data and therefore cannot be used to represent an accurate cross section of the GB poultry industry. The database does however give an accurate representation of the potential links between premises that do fit the above criteria.

**Figure 1:**
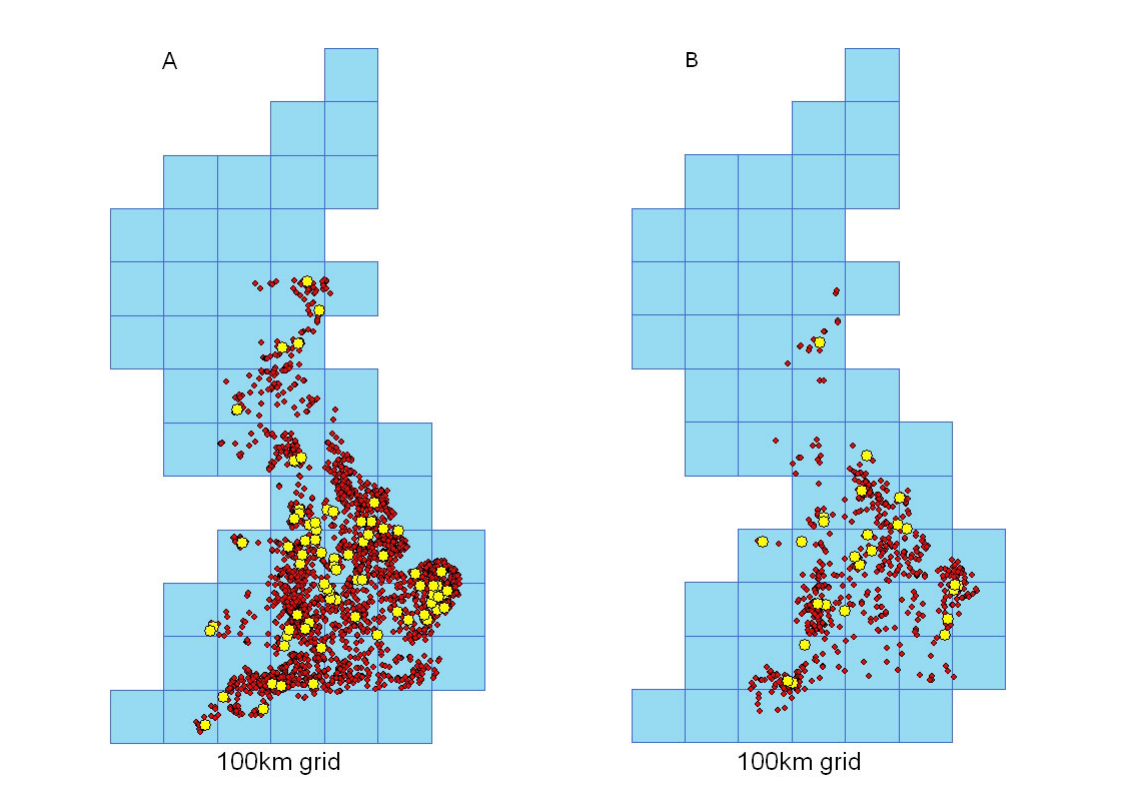
Poultry premises (red) using A) Slaughterhouses (yellow), and B) Catching companies (yellow).

Of the premises included in the sample, 2973 premises use identifiable slaughterhouses (Figure 1A), 707 premises use identifiable catching companies (Figure 1B) and 1016 have company affiliations. Premises associated with a third party (slaughterhouse, catching company or multi-site company) are assumed to be potentially connected to any other premises associated with the same third party. Data are stored in the poultry network database. Data in the network database were compared with the GB Poultry Register (GBPR) to look for biases. The GBPR contains information about all poultry premises housing 50 or more birds.

### Industry demographics

**Figure 2:**
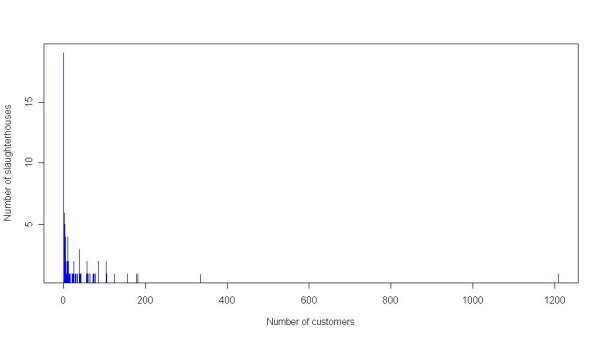
Slaughterhouse customers (poultry premises) against the frequency of slaughterhouses with that many customers.

Based on a list of abattoirs licensed to slaughter poultry, maintained by the Food Standards Agency (FSA), it is estimated that over 90% of slaughterhouse through-put (by number of birds) is accounted for in the network database. Further, the spatial distribution of slaughterhouses and customers in the network database is similar to that of the overall GB population [Jason Gittins, pers. comm.]. However, the number of premises in the network database that are recorded as having sent birds to slaughter represents only 13% of the number of premises in the GBPR, implying that larger premises are better represented in the network database. The number of customers per slaughterhouse ranges from one to over one thousand customers, with a median of ten customers (Figure 2).

**Figure 3:**
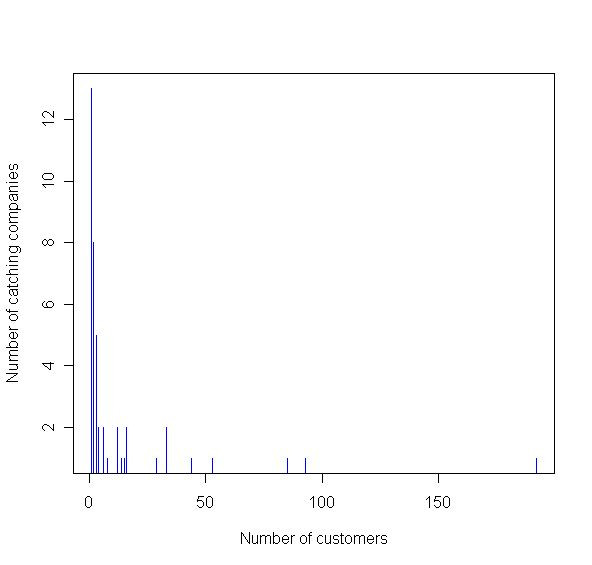
Catching company customers (poultry premises) against the frequency of catching companies with that many customers.

**Figure 4:**
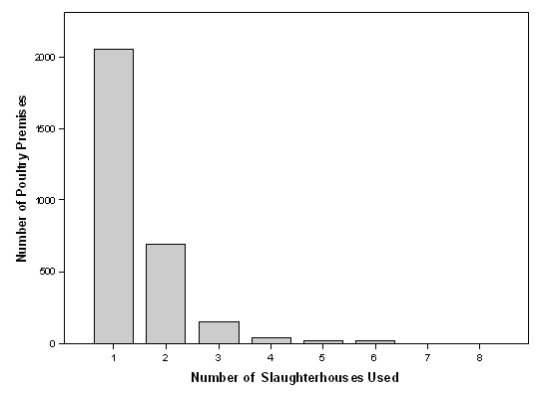
The number of Slaughterhouses used per poultry premises against the frequency of poultry premises.

Over 50% of the identified premises that use catching companies are comprised of housed broilers (reared for meat) chickens. This corresponds with expectations that over 50% of catchers are involved in the broiler chicken sector [29], though the database is not confirmed to be complete. The database contains information from 25 individual catching companies. It has been reported that 8 specialist catching companies, included in the database, account for 50% of the chicken sector [Jason Gittins, pers. comm.]. As all known large (catching from many premises) catching companies are included in the database, it is assumed that catching companies that are not included in the database are small companies that do not necessarily catch from many premises. For end of lay hens, most of the larger producers use specialist companies including those currently in the database. The number of catching company customers (over 45 catching company sites) ranges from one to 192 customers (Figure 3). Of the premises visited by catching companies, fewer than 2% of premises (12 of 707 premises) are recorded as using more than one catching company, with no poultry premises using more than two different catching companies. The overlap between slaughterhouses is much greater, with over 30% of premises (913 of 2973 premises) sending birds more than one slaughterhouse (Figure 4).

A total of 1003 (98.7%) of multi-site premises were matched to the GBPR. For multi-site sources, premises were identified as being part of a multi-site company via questionnaires completed by both individual premises and company representatives. The legislation requires the owners of all premises housing 50 or more birds, kept for commercial purposes, to register their poultry on the GBPR. We assume that the multi-site data gives a good representation of the population. The number of premises per multi-site company ranges from two to 113, with the median number of premises in a multi-site company being six.

### Contact structures

The contact structure can be viewed as a social network of potentially infectious links amongst premises, these links are defined by associations via one or more of the four potential routes of interaction. Transmission networks can then be generated by assuming a probability of transmission, *p*, associated with those links, and then selecting or discarding them based on whether a random number, chosen from a uniform distribution from zero to one, is above or below that value *p*. The transmission networks can then be grouped into "components" wherein all premises within a component can be linked to any other member. In a transmission network, the size of a component is therefore an estimate of the size of an epidemic should any member of that component become infected, in the absence of intervention. A giant component (GC) is the largest component, and therefore the size (calculated as the number of nodes) of the GC represents an estimate for the upper bound for the potential size of an epidemic that starts with a single infected premises and in the absence of further intervention that would alter the network structure. While intervention will occur as soon as AIV is detected and therefore an epidemic of this size is unlikely to ever be reached, nevertheless in other similar scenarios, drastic increases in GC size has previously been shown to be a good indicator of when a population is vulnerable to a serious epidemic [19].

### Contact structure analysis – worst-case scenario

**Figure 5:**
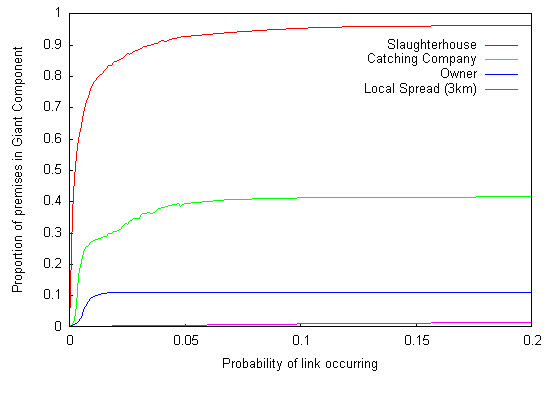
Proportions of premises contained in the Giant Component (GC) for each transmission route, for probability of a link occurring between connected premises varied between p = 0 and p = 0.2.

**Figure 6:**
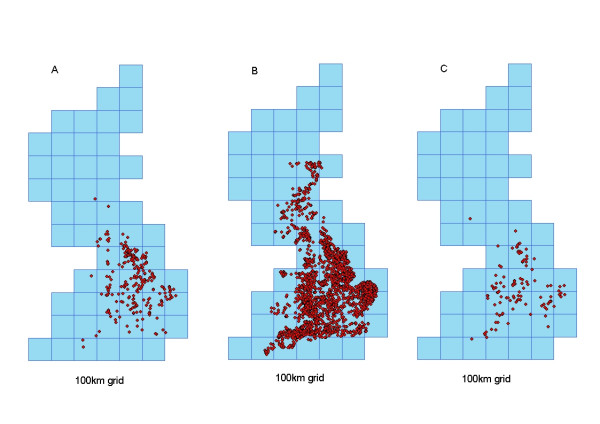
Location of poultry premises contained within the GC for premises connected by A) Catching Company, B) Slaughterhouse C) Owner. With between species transmission. Premises plotted on a 100 km Grid, for probability of a link occurring between two premises equal to 0.2.

In order to gain an understanding of different parts of the industry, we have considered the four contact structures separately. When all contact structures are combined, the GC constantly covers the majority of premises thus making it difficult to determine how the different contact structures affect the potential for large epidemics. Links are established between all premises using the same slaughterhouse, catching company, owner or between premises that are geographically close (within 3 km of each other). By assuming that all premises within a given contact structure are potentially connected, we can determine the worst-case scenario, where no interventions are made over time. Based on an analysis of movement data between farms using catching companies and sending birds to slaughter [Dent, unpub.] links between premises are not directional. As the probability of disease transmission occurring was increased, the proportion of premises in the transmission network GC increased until a maximum of approximately 2870 (97%) of premises linked by slaughterhouses, 295 (42%) of premises linked by catching companies, 113 (11%) of premises linked by owner and 111 (2%) of premises linked by being geographically close to one another, was reached for small probabilities of a link occurring (Figure 5). The GC for the slaughterhouse network noticeably covered a wider area and was denser than the GCs for the catching company and owner networks (Figure 6).

### Network analysis – removal of key players

A "key player" can be defined as an individual member of the network whose removal has a major impact on reducing the size of a potential epidemic, or equivalently the size of the GC [30]. Here, key players are premises that link otherwise separate components, such as poultry premises that are the only links between companies using separate slaughterhouses or the slaughterhouse linking the most premises.

The three premises that use the most slaughterhouses (two using seven different slaughterhouses and one using eight) were removed from the network. However, the size of the GC remained close to 2870 showing that this had only a minor effect on the worst-case epidemic. Removal of premises from the network represents movement bans that are imposed during an outbreak. These results suggest that targeting surveillance or imposing movement bans at highly connected premises alone would not necessarily have a large impact on the potential size of an epidemic. Removal of the slaughterhouse with the largest number of customers (1208 customers) resulted in the number of premises sending birds to slaughter to fall by 883 (29%) premises, suggesting that if a large slaughterhouse were to be involved in an outbreak, there is some justification in spending money on forward and backward tracings as well as the local protection and surveillance zone that is required.

### Sensitivity analysis

#### Relative importance of different contact structures

The proportion of premises not connected to any other premises i.e. with zero degree, is significantly less (p-value < 0.01 (ANOVA)) for premises connected by slaughterhouse than for those connected by catching company or by owner. Furthermore, as the probability of a link occurring increases, the proportion of zero-degree premises drops significantly more quickly for premises connected by slaughterhouses (p-value = 0.028 (ANOVA)) than for premises connected by other potential transmission routes. This implies that individual premises are much more likely to be connected to other premises via the slaughterhouse route than any other route, and as the probability of spread via each route increases, the number of premises connected to at least one other premises increases more quickly for premises linked via slaughterhouses than for other routes. Although this suggests slaughterhouses are relatively more important than catching companies and owners in terms of the number of premises that they are likely to connect, it does not imply that they are more likely to spread disease, as these results do not take into account the quantitative probability of transmission via each route.

#### The role of premises using multiple slaughterhouses

According to the available contact data, the owners of poultry premises send birds to up to eight different slaughterhouses though according to Gittins [ADAS, pers. comm.] only a small number of single species farms would truly send birds to more than one slaughterhouse at any one time. When the number of slaughterhouses associated with a premises is restricted to one, the maximal size of the GC in the slaughterhouse transmission network is reduced to 970 (32.6%) premises. By reducing the number of slaughterhouse that premises can send birds to, we are able to comment on the impact that premises housing multiple species have on the potential for disease transmission.

#### Treating multi-species sites as separate epidemiological units

In the preliminary analyses of the contact structures described in this study, we made an assumption that catching teams within a catching company are able to catch from any farm that is associated with that company, and birds can be sent from a poultry premises to any slaughterhouse associated with that premises. This assumption may over estimate the number of contacts within the industry as catching teams and slaughterhouses often process only one species and/or production type e.g. spent chicken layers and meat chicken are not necessarily processed at the same slaughterhouse. Under the assumption that different species cannot be connected by slaughterhouse or catching company, multi-species sites can be treated as separate epidemiological units that are connected by location and company.

**Figure 7:**
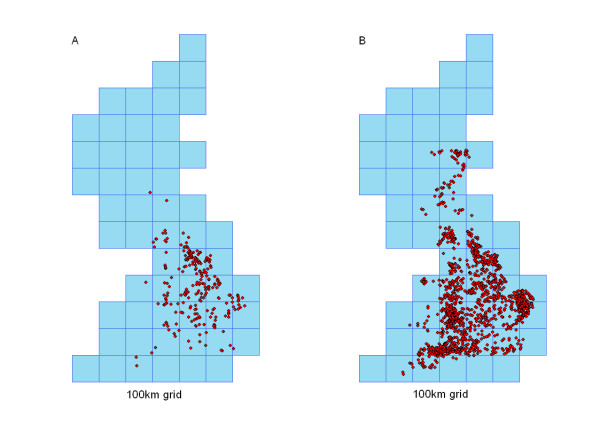
Location of poultry premises contained within the GC for premises connected by A) Catching Company, B) Slaughterhouse. With no between species transmission. Premises plotted on a 100 km Grid, for probability of a link occurring between two premises equal to 0.2.

Properties of the contact structures are sensitive to the assumption that connections are likely to occur between different species and between different production-types within a species. This is shown by the fall in the size of the GC to 1603 (53.9%) of premises connected by slaughterhouse and to 102 (14.4%) of premises connected by catching company when between species transmission is not assumed (Figure 7). Furthermore, when we assume that different species are not processed together, we see a drop in the degree (number of links to other premises) of each premises. The mean degree size falls from 18.12 to 8.33 links and from 504.95 to 28.64 links for premises connected by catching companies and slaughterhouses respectively, when no between-species transmission occurs via these routes. As restricting contact between different species causes the number of premises with zero-degree to rise and the size of maximum degree to fall (Table 2), we can expect the basic reproduction number R0 for a disease to also fall if between species transmission can be controlled or prevented on multi-species sites.

**Table 2:** Difference in degree size for between species and no-between species transmission

	Slaughterhouse: Between species transmission	Slaughterhouse: No between species transmission	Catching company: Between species transmission	Catching company: No between species transmission
Proportion of zero-degree epidemiological units	0.101	0.310	0.789	0.851

Largest degree	1457	700	223	107

Numbers correspond to number of epidemiological units, and not number of premises. By splitting multi-species premises into separate epidemiological units, the number of units associated with catching companies and slaughterhouses rise to 825 and 3418 respectively.

#### Defining a maximum distance that any catching team can travel between two poultry premises

**Figure 8:**
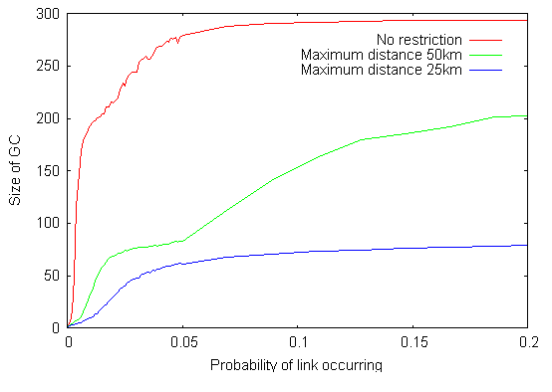
The effect on the number of premises in the GC of restricting the distance that catching companies move between premises to a) no restriction (red), b) 50 km restriction (green) and c) 25 km restriction (blue).

**Figure 9:**
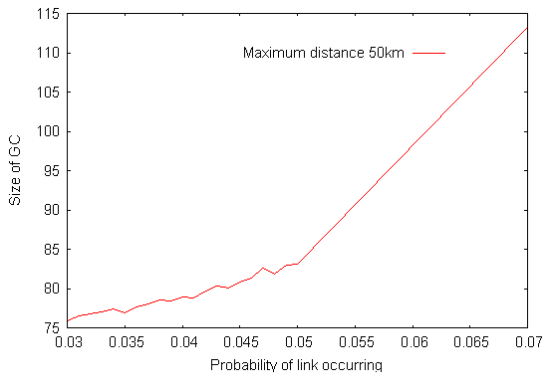
Evidence of geographic isolation for transmission networks generated when the probability of transmission via catching companies passes through *p *= 0.05. Size of the GC at just below and just above the transition.

Some catching teams operate over broad regions of GB [29]. This may occur when the birds to be caught require specialist catching skills, such as for turkeys because of their size and weight. In order to determine if the area over which a catching team operates affects the contact structure of the poultry network, we restricted the distance over which a team could operate i.e. the distance that any one catching team within a company can physically travel between farms. Radii of different sizes were therefore used around premises, only allowing links to occur with other premises within the radii. Restricting this distance reduced the size of the GC from 295 (41.7%) of premises for no restrictions to 229 (32.4%) for a restriction of 50 km and 84 (11.9%) of premises for a restriction of 25 km (Figure 8). Evidence of some geographic isolation is found when the maximum distance is restricted to 50 km; for transmission networks generated when the probability of transmission via catching companies passes through *p *= 0.05, the number of premises visited by a catching team, on average, takes a rapid jump from below ten to more than 13 premises (Figure 9).

## Discussion

By combining available data associated with prior outbreaks of poultry diseases associated with faeco-oral transmission routes (Table 1) we have constructed possible contact structures within the poultry industry in GB based on associations amongst poultry premises using the same slaughterhouses, catching companies, and belonging to the same multi-site companies. We have also included environmental spread in our analyses by assuming that disease can be transmitted between premises that are geographically close to each other.

Outbreak data from the Netherlands shows that local transmission of HPAI played an important role in the 2003 epidemic [13,31]. Boender (2007) suggests that epidemic spread is only possible in poultry dense areas of The Netherlands [31]. In the analyses shown here, only 2% of premises were connected, in the worst case scenario, via local transmission within 3 km of an infected premises, suggesting that the British poultry industry is not densely enough populated for local transmission of the HPAI virus which devastated the Netherlands in 2003.

If one assumes that using the same slaughterhouse company implies a potential link, up to 97% of premises sending birds to slaughter are potentially connected, which could translate to almost the entire poultry industry, assuming that most commercial premises do not slaughter their birds on site. In contrast, only 42% of premises using catching companies and 11% of premises belonging to multi-site companies are potentially linked. Although this suggests that slaughterhouses potentially link the largest number of premises and therefore have the highest potential for widespread dissemination of virus, should virus transmit via this route, the size of the GC was highly sensitive to the number of slaughterhouses used per premises. It seems unlikely that over 30% of premises truly send birds to more than one slaughterhouse, as indicated in the network database. It is possible that when slaughterhouses were asked to provide a list of customers, some premises were listed that are no longer active customers. This could result in an overestimate in the number of slaughterhouses used per premises. This suggests that the structure of the industry may be very dynamic, with premises changing their potential interactions regularly. Thus regular updating of the database would be necessary if it is to be used for contact tracing purposes. Further, in the absence of contact data from an AIV epidemic in GB, we do not know the parameter values that should be applied to each transmission mechanism and should therefore take care when comparing outputs, and it is generally believed that catching company teams, for example, are a more likely mode of transmission than slaughterhouses (cf. table 1). We also note that different research groups have approached this problem in different ways. Truscott *et al *(2007) [8] group movements of people and equipment together and assume a constant, density-independent contact rate between premises, where-as Sharkey *et al *(2007) [17] do not incorporate the movement of catching companies in their models but do consider the probability of transmission via slaughterhouses to be always greater than that of company movements for example. In adopting either approach, slaughterhouses remain the most important contact mechanism in this analysis in terms of the number of premises that may become infected. Further data collection is required to determine why the owners of poultry do not necessarily use local catching companies and slaughterhouses, and whether putting a smaller limit on the distance that live poultry can be transported would be a feasible standard for the industry to set.

The contact structures observed here are well connected with a high number of links between premises. This occurs because we have made the assumption that all premises using the same slaughterhouse, catching company or belonging to the same multi-site premises are potentially all connected. This means that targeting surveillance at the premises that use the most number of slaughterhouses, in particular, will not be beneficial in preventing or controlling an epidemic as there are other premises, using more than one slaughterhouse, that are able to keep the connectedness of the contact structure. We have shown that removal of the largest slaughterhouse greatly reduces the number of premises that are connected. While one cannot remove a slaughterhouse from the industry in real terms, one can target surveillance, through forward and backward tracings, at all of the premises that have had recent contact with the slaughterhouse. By ensuring that there is no infection passing through the largest slaughterhouse, we can be sure that at least 22% of premises that send birds to slaughter are not transmitting disease via this mechanism.

Multi-species sites are also potentially important, should transmission between species on a site be likely, as they can act as a bridge between different sectors of the poultry industry. Operating on a species-specific basis at the slaughterhouse and by the catching company can reduce the risk of a large epidemic, by reducing the number of potential contacts made between separate epidemiological units. This in turn reduces the R0 of a disease, making control more manageable. Housing multiple species on the same site so that species have the potential for interaction, either by being housed in the same building or through having access to the same feeding or watering ground for example, may also pose problems at the farm level as a result of the differences in species susceptibility to AI viruses. Ducks for example are able to carry both LPAI and HPAI virus without showing any clinical signs [2,32]. Although outbreaks of HPAI in commercial ducks are rare, the ability of ducks to survive infection can increase the time to detection of an outbreak, and hence the number of premises potentially infected with an AI virus. This is particularly dangerous for premises housing ducks and chickens or turkeys, as ducks can shed high doses of the virus without any early warning signs. While further investigation into the range of values of within flock transmission is important, these analyses underline the value of good biosecurity at premises level to limit transmission across species within premises.

Biosecurity measures are not directly accounted for in this analysis. Under good biosecurity measures, connections between species and connections between premises, over which disease can transmit, can be broken. Although biosecurity levels are difficult to measure, they can be represented here by a reducing the probability of an infectious link occurring between connected premises. This would result in a reduction of the number premises in the GC. The real risk of disease transmission through movements of people, vehicles and equipment should be investigated further, so that the impact of biosecurity at both the farm and slaughterhouse level can be simulated.

## Conclusion

We have used potential transmission routes of poultry diseases, to identify potential contact structures within the poultry industry in GB over which AIV may transmit. Few premises are connected as a result of being geographically close to one another, which reduces the concern for local spread of AIV, and limits the validity of applying data from the 2003 outbreak in the Netherlands to the GB situation.

Connections through slaughterhouses potentially links surprisingly large numbers of premises, over long distances. Further work as to whether these potential connections represent real risk, or are just an artefact of the data, must be investigated. Should it prove true, surveillance should be targeted at those premises connected to the largest slaughterhouse in order to prevent disease spreading to a large number of premises. As reducing the distance that catching companies travel between premises reduces the number of premises that are potentially connected, we also suggest controlling wide dissemination of disease by encouraging premises to use local catching companies and slaughterhouses.

If between species transmission occurs, then this has implications for the potential for large epidemics as multi-species sites may play an important role in the connectivity of otherwise separate sectors of the poultry industry, though expert opinion suggests that they are only likely to interact at the local level. This makes a difference to the maximum number of premises that may be connected and hence gives rise to the importance of further investigations into this area.

## Methods

### Literature review

A literature review was undertaken in order to research the British poultry industry and to identify potential between farm transmission routes of AIV. The results were collated with expert opinion and categorised into sub-populations within the poultry industry that may connect poultry premises.

### Construction of contact structures

A series of epidemiological contact structures were constructed in which commercial poultry premises are linked by potentially infectious contacts. The potential contacts between premises were informed by a NEEG/CERA (National Epidemiology Emergency Group/Centre for Epidemiology and Risk Analysis, Defra) data collection exercise, in which slaughterhouses and catching companies were approached to provide a list of the premises from which they collect birds and the species involved, and a sample of single and multi-site companies were sent a questionnaire on which they were asked for details about the frequency and type of movements from their premises. In addition, the poultry register (GBPR) data provided details on the location and species numbers in each poultry premises in GB housing more than 50 birds. The data in the network database were compared to the GBPR as well as to data obtained from the Food Standards Agency and expert opinion [Jason Gittins, Howard Hellig, Ian Brown] in order to determine how representative the network database is of the contact structures being analysed.

Potentially infectious links between premises could occur either as a result of premises:

1. using the same catching company, or

2. using the same slaughterhouse, or

3. belonging to the same multi-site company, or

4. environmental spread within a 3 km radius of poultry premises.

We assumed that premises could be linked by catching company or multi-site companies by the direct movements of people (catching teams or company personnel), vehicles or equipment between poultry premises. For slaughterhouses, connections between premises can occur when slaughterhouse vehicles and equipment are used on multiple premises to collect birds. Vehicles may visit multiple premises en-route to the slaughterhouse, possibly connecting farms and transmitting infection, or they may return to a slaughterhouse between visits to premises. The use of slaughterhouse vehicles and equipment in the transportation of birds to slaughter can connect farm to slaughterhouse to farm, or farm to farm to slaughterhouse.

A radius of 3 km was chosen to be limit for environmental transmission, based on small probabilities of transmission of AIV via this route [31]. A 3 km radius is also the radius of the protection zone put around infected premises in GB, during an outbreak situation.

Under the assumptions made, maximum connectivity between premises is represented in the contact structures. A potential epidemic supported by such a contact structure could be considered the worst-case scenario.

### Contact structure analysis

Having identified the contact structures to be analysed, the number of nodes and links were calculated for each type of potentially infectious link. The four contact structures were analysed using a simulation programme. The programme, written in C language, uses Tarjan's algorithm [33] to find the largest component within the given contact structure such that any premises in that component can be reached directly, or indirectly, by any other premises. This output represents an upper limit to the size of a potential epidemic that may occur as a result of transmission of disease via a particular transmission mechanism. The probability of a link occurring between two premises, which was varied between zero and one, represents the probability of transmission of disease.

Initially, the worst-case scenario was considered, where all links result in potentially infectious contacts. In order to test the sensitivity of the results to the assumption that all links result in potentially infectious contacts, the effects that removing key-players from the network have on the size of the GC was considered, as well as the effect of restricting poultry premises to the use of only one slaughterhouse, chosen at random from a list of slaughterhouses used by the premises.

### Sensitivity analysis

It should be noted that the sensitivity analysis performed here is used to investigate the impact of changes (e.g. restrictions on distance travelled by catching teams) on the properties of contact structures. By considering certain properties of the contact structures, we can investigate the relative sensitivity of the contact structure properties under different scenarios. In order to determine the relative importance of each contact structure, we consider how the degree distribution (the number links per premises) varies as the importance of the contact structure changes. The "importance" can be varied by varying the probability of a link occurring between two premises within the contact structure.

The sensitivity of the GC to the following scenarios was explored and areas for further data collection identified:

1. Limiting the number of slaughterhouses to one per premises: Slaughterhouses generally do not slaughter multiple species. By limiting the number of slaughterhouses used per premises, we were able to determine the effect that premises housing multiple species have on the connectivity of the contact structure represented by slaughterhouses, as such premises are the ones likely to be sending birds to multiple slaughterhouses. Where premises are recorded as sending birds to multiple slaughterhouses, one slaughterhouse was chosen at random.

2. Treating multi-species sites as separate epidemiological units: Different species housed on the same site are treated as separate epidemiological units, categorised into the five principal sectors of the British poultry industry: meat chicken, commercial layer, turkey, duck and goose industries [34]. We assume that between-species transmission can only occur via local (i.e. short distance) spread and not via slaughterhouse or catching company transmission. Under these assumptions, the impact that the possibility of cross-species contamination, particularly on multi-species farms, could have on the potential for disease transmission was explored.

3. Imposing a maximum distance that any catching team can travel between two poultry premises: The Euclidean distance between two potentially connected premises was calculated, and the link could only result in disease transmission if two premises are within a given distance of each other (distances of 25 km and 50 km were tested). This analysis revealed the importance of obtaining more detailed catching company and slaughterhouse data.

## Abbreviations

ADAS: Agricultural Development and Advisory Service; AIV: avian influenza virus; CERA: Centre for Epidemiology and Risk Analysis; Defra: Department for Environment, Food and Rural Affairs; FSA: Food Standards Agency; GB: Great Britain; GC: Giant component; HPAI: High-pathogenic avian influenza; LPAI: Low pathogenic avian influenza; NEEG: National Epidemiology Emergency Group; UK: United Kingdom.

## Authors' contributions

MA is responsible for the conception of the study. MA, RK, IK and JD contributed to the design of the study. JD and IK carried out the analyses of the data. Interpretation of the results was undertaken by all authors. The manuscript was written by JD with comments from all authors.
